# Efficient 3D kernels for molecular property prediction

**DOI:** 10.1093/bioinformatics/btaf208

**Published:** 2025-07-15

**Authors:** Ankit, Sahely Bhadra, Juho Rousu

**Affiliations:** Department of Data Science, Mehta Family School of Data Science and Artificial Intelligence, Indian Institute of Technology Palakkad, Kanjikkode, Palakkad, Kerala 678623, India; Department of Data Science, Mehta Family School of Data Science and Artificial Intelligence, Indian Institute of Technology Palakkad, Kanjikkode, Palakkad, Kerala 678623, India; Department of Computer Science, Aalto University, Espoo 02150, Finland

## Abstract

**Motivation:**

This paper addresses the challenge of incorporating 3-dimensional (3D) structural information in graph kernels for machine learning-based virtual screening, a crucial task in drug discovery. Existing kernels that capture 3D information often suffer from high computational complexity, which limits their scalability.

**Results:**

To overcome this, we propose the 3D chain motif graph kernel, which effectively integrates essential 3D structural properties—bond length, bond angle, and torsion angle—within the three-hop neighborhood of each atom in a molecule. In addition, we introduce a more computationally efficient variant, the 3D graph hopper kernel (3DGHK), which reduces the complexity from the state-of-the-art $O(n^{6})$ (for the 3D pharmacophore kernel) to $O(n^{2}(m+\log(n)+\delta^{2}+dT^{6}))$. Here, *n* is the number of nodes, *T* is the highest degree of the node, *m* is the number of edges, δ is the diameter of the graph, and *d* is the dimension of the attributes of the nodes. We conducted experiments on 21 datasets, demonstrating that 3DGHK outperforms other state-of-the-art 2D and 3D graph kernels, but it also surpasses deep learning models in classification accuracy, offering a powerful and scalable solution for virtual screening tasks.

**Availability and implementation:**

Our code is publicly available at https://github.com/SantaAnkit/Efficient-3D-kernels-for-molecular-property-prediction.git.

## 1 Introduction

In drug discovery, virtual screening is a rapid and cost-effective method that is used to evaluate a large number of potential drug candidates. It involves two main strategies: ligand-based drug design (e.g. ligand similarity) and structure-based drug design (e.g. ligand docking). Structure-based methods require 3D data of the target protein to determine the binding affinity of the ligand. When such data are unavailable, ligand-based methods are used, leveraging the idea that structurally similar ligands tend to have similar activity. Machine learning aids ligand-based virtual screening by treating the problem as graph classification, where ligands are modeled as graphs, and algorithms predict their interaction with target proteins. The two key approaches are kernel-based methods and neural network-based methods. In this research, graph kernel-based methods are preferred due to their superior performance with small datasets, as deep learning models typically underperform when data are scarce and expensive (Zhao *et al.* 2022).

To accurately measure the similarity between ligands, it is crucial to understand their 3-dimensional (3D) spatial arrangement, which helps reveal binding mechanisms and predict binding affinities. Graph kernels are commonly used to determine structural similarity between ligands. However, while 2D graph kernels are well-studied, incorporating 3D structural information in graph kernels remains challenging due to higher computational complexity. As a result, many existing graph kernels fail to capture important spatial features such as bond lengths, bond angles, and torsion angles of ligands (Acharya *et al.* 2011, Ganea *et al.* 2021, Ragoza *et al.* 2022).

3D ball kernels (Wang and Scott 2005) and torsion kernels (Qiu *et al.* 2007) are 3D kernels defined for proteins. The 3D ball kernel (Wang and Scott 2005) visualizes proteins as 3D spaces filled with various balls but lacks key geometric features (Vaidehi and Jain 2015, Glasser 2020) such as bond angles and torsion angles. The torsion kernel (Qiu *et al.* 2007) partially addresses this by incorporating torsion angle information through a spectral kernel (Leslie *et al.* 2002), yet it omits bond length and bond angle data. These kernels are not defined for ligands, and moreover, they lack capturing all 3D geometric information, hence it is important to define a 3D kernel for small molecules or ligands (Acharya *et al.* 2011, Ganea *et al.* 2021, Ragoza *et al.* 2022).

Pharmacophores (Mahé *et al.* 2006), defined as 3D configurations of atoms contributing to a drug’s biological activity, also face limitations. They fail to include torsion angles since pharmacophores typically involve three distinct nodes, while torsion angles require four consecutive nodes. Moreover, computational complexity becomes a concern when handling large datasets. The matrix-based pharmacophore kernel (PK) requires $O(\frac{n^{6}}{k^{3}})$ time for two graphs with *n* nodes and *k* atom classes, making it inefficient for large-scale applications. Thus, there is a need for a new graph kernel that effectively incorporates bond length, bond angle, and torsion angle information while maintaining computational efficiency.

In our research, we focus on incorporating comprehensive 3D structural information bond lengths, bond angles, and torsion angles of molecules (ligands) into a scalable and optimized kernel framework. To this end, we introduce a 3D chain motif graph kernel (c-MGK), which effectively captures all critical 3D structural details using distance-based measures. Additionally, we present an improved, faster variant called the 3D graph hopper kernel (3DGHK), designed to achieve the same goals with improved efficiency. Both kernels are translation and rotation invariant.

We evaluated our kernels on 21 datasets, comparing their performance with existing 2D and 3D kernels, and graph neural network. The results demonstrated that our kernels outperformed others in terms of classification accuracy, while the 3DGHK achieved superior computational efficiency compared to other 3D kernels. Key contributions are summarized below.

- We introduce a novel 3D c-MGK that integrates key 3D structural features bond length, bond angle, and torsion angle to better capture molecular interactions.
- We theoretically demonstrate that torsion angles can be expressed using six pairwise distances between any four consecutive atoms, providing a more efficient way to encode torsion information.
- Instead of defining the GHK based on motifs, we redefine the node kernel of the GHK as a 3D node kernel, ensuring that it directly incorporates 3D structural information.
- We show that the c-MGK takes $O(n^{2}T^{6}(nT^{4}+\text{log}(nT^{3})+\delta^{2}+d)$ time. Here, *n* is the number of nodes, *T* is the highest degree of the node, *m* is the number of edges, δ is the diameter of the graph, and *d* is the dimension of the attributes of the nodes. This complexity is reduced to $O(n^{2}(m+\text{log}(n)+\delta^{2}+dT^{6}))$ for the 3DGHK. While 3DGHK is faster than existing 3D kernels, it remains slower than 2D kernels due to the additional complexity of processing 3D structural information.
- Our proposed 3DGHK consistently outperformed other state-of-the-art 2D and 3D kernels and deep learning methods in terms of classification accuracy except Weisfeiler–Lehman kernel (WLK). Additionally, it achieved significantly better computational efficiency compared to other 3D kernels.

## 2 Previous work

Over the years, numerous graph kernels have been developed to capture structural similarities between graphs (Kriege *et al.* 2020). Many of these kernels (Giannis *et al.* 2021) decompose graphs into smaller components to facilitate comparison. One prominent approach is the R-convolution kernel (Haussler 1999), which decomposes graphs into substructures and computes the overall kernel value by summing the similarities between corresponding substructures. This framework underlies the majority of existing graph kernels (Giannis *et al.* 2021).

The random walk kernel (RWK) (Gärtner *et al.* 2003) measures graph similarity by counting matching random walks within different graphs, with an initial time complexity of $O(n^{6})$. To improve efficiency, Vishwanathan *et al.* (2010) proposed kernel using product graphs, which perform random walks on a composite graph instead of individual ones, reducing the time complexity to $O(n^{3})$. In contrast, Shervashidze *et al.* (2009) introduced a graphlet-based kernel, which counts specific-sized subgraphs (graphlets) to assess graph similarity. While effective, this approach is computationally intensive, particularly for graphlet sizes greater than 5, making it less feasible for larger graphs.

As graph sizes increase [e.g. (n≥100), many kernels such as the RWK ($O(n^{3})$], shortest path kernel (SPK) ($O(n^{4})$) (Borgwardt and Kriegel 2005), and graphlet kernel (Shervashidze *et al.* 2009) suffer from high computational complexity. To address this issue, Shervashidze *et al.* (2011) introduced the WLK, based on the WL isomorphism test. This kernel efficiently compares graphs by iteratively relabeling node neighborhoods using a nonlinear relabeling function, thereby creating representation vectors for each graph, which can be used for similarity comparisons. When the node labels are in 1D space, the WL kernel performs better and does not generalize to graphs with continuous vector node attributes.

Additionally, several graph kernels are based on paths, including the SPK (Borgwardt and Kriegel 2005), cyclic, and tree kernels (Horváth *et al.* 1999), and GHKs (Feragen *et al.* 2013). These kernels capture graph similarity by identifying common patterns, such as shared paths or substructures. The SPK measures graph similarity by counting common shortest paths between similar nodes. However, its time complexity of $O(n^{4})$ makes it inefficient for large graphs. The GHK (Feragen *et al.* 2013) is designed for graphs with continuous node and edge attributes. It defines similarity based on the nodes encountered along the shortest paths between any two nodes. This approach significantly reduces the runtime complexity from $O(n^{4})$ to $O(n^{2}(m+\log(n)+\delta^{2}+d))$, where *m* is the number of edges, δ is the diameter of the graph and *d* is the dimension of node attributes. This reduction in complexity makes the GHK more practical for large-scale graph comparison tasks.

Despite their widespread use and effectiveness, all of the aforementioned kernels primarily focus on capturing 2D topological properties such as the adjacency matrix, node attributes, and edge attributes. However, they lack the capability to incorporate 3D structural information, which is crucial in applications involving molecular structures. To our knowledge, several 3D kernels have been proposed, including the torsion kernel (Qiu *et al.* 2007), the 3D ball kernel (Wang and Scott 2005), and the PK (Mahé *et al.* 2006), each of which integrates 3D information differently. The torsion kernel represents a chain sequence as a series of torsion angles formed by consecutive four C−α atoms. To build the similarity matrix, these torsion angles are discretized into *n* bins, each assigned a distinct label. The spectral kernel (Leslie *et al.* 2002) is then applied to these discrete features. While effective for capturing torsion angles, the torsion kernel may not fully describe the molecule’s spatial arrangement. The 3D ball kernel (Wang and Scott 2005) models proteins by placing virtual balls around each amino acid in a 3D space. The radius of each ball, set as a hyperparameter, determines its size, and the kernel is defined by comparing the similarities of these balls across different proteins. Although this approach captures spatial proximity, it does not incorporate key structural details like bond angles and torsion angles. The PK (Mahé *et al.* 2006) defines a three-point pharmacophore, using the 3D positions of atoms and their labels to compute the similarity between pharmacophores. By leveraging the properties of triangles formed by three atoms, this kernel effectively captures distance and bond angle information, it does not explicitly incorporate torsion angles, limiting its ability to represent complex molecular conformations comprehensively.

## 3 Novel 3DGHK

### 3.1 Background and notations

In this paper, we use the notation G={V,E,Z,l} to show 3D graphs and G={V,E} to show a 2D graph without 3D coordinate information. Here, V=V is the set of vertices, E=E is the set of edges, Z is the set of 3D coordinates of the vertices, and l:V∪E→L is the labeling function for the vertices and edges of G. Note that L can be any label, either discrete or continuous. The set of vertices in the neighborhood of v is given by N(v)={u∈V:(u,v)∈E}.

### 3.2 Graph hopper kernel

Consider a graph G(V,E) with some labeling function l:V→L which maps each node to its corresponding attributes. Given two nodes u,v∈G the path π between them is defined by the sequence of nodes that traversed in between. $\pi =[v_{1},v_{2},...,v_{n}]$ where $v_{1}=u,v_{n}=v$ and $(v_{i},v_{i+1})\in E,\forall i=1,2,\ldots ,n-1$. The GHK (Feragen *et al.* 2013) between two graphs *G* and $G^{\prime }$ is defined as the sum of the path kernels as follows.


(1) $$K(G,G^{\prime })=\sum_{\pi \in P}\sum_{\pi^{\prime }\in P^{\prime }}k_{p}(\pi ,\pi^{\prime })$$

*P* and $P^{\prime }$ are the family of paths in their respective graphs *G* and $G^{\prime }$. This path kernel $k_{p}(\pi ,\pi^{\prime })$ can be seen as the sum of the node kernel on the vertices that came into the same place while hopping, that is


$$k_{p}(\pi ,\pi^{\prime })=\{\begin{array}{cc} \sum_{j=1}^{\left|\pi \right|}k_{n}(\pi ,\pi^{\prime }), & \text{if}\;\pi =\pi^{\prime }\\ 0, & \text{otherwise} \end{array}$$

Where |π| defines the discrete length of node in path π. The diameter of a graph δ(G) of *G* is the maximal number of nodes in the shortest path in *G*. Therefore,


(2) $$\begin{array}{l} K(G,G^{\prime })=\sum_{v\in V}\sum_{v^{\prime }\in V^{\prime }}w(v,v^{\prime })k_{n}(v,v^{\prime })\\ w(v,v^{\prime })=\sum_{j=1}^{\delta }\sum_{i=1}^{\delta }\#\{(\pi ,\pi^{\prime })|\pi (i)=v,\pi^{\prime }(i)=v^{\prime },\\ |\pi |=|\pi^{\prime }|=j\}=<M(v),M(v^{\prime })> \end{array}$$

where M(v) is a δ×δ matrix whose $ij^{th}$ entry counts how many times *v* appears at the $i^{th}$ coordinate of a shortest path in G of discrete length *j*, and $\delta =\max\{\delta (G),\delta (G^{\prime })\}$.

### 3.3 3D structural information

**Figure 1.**
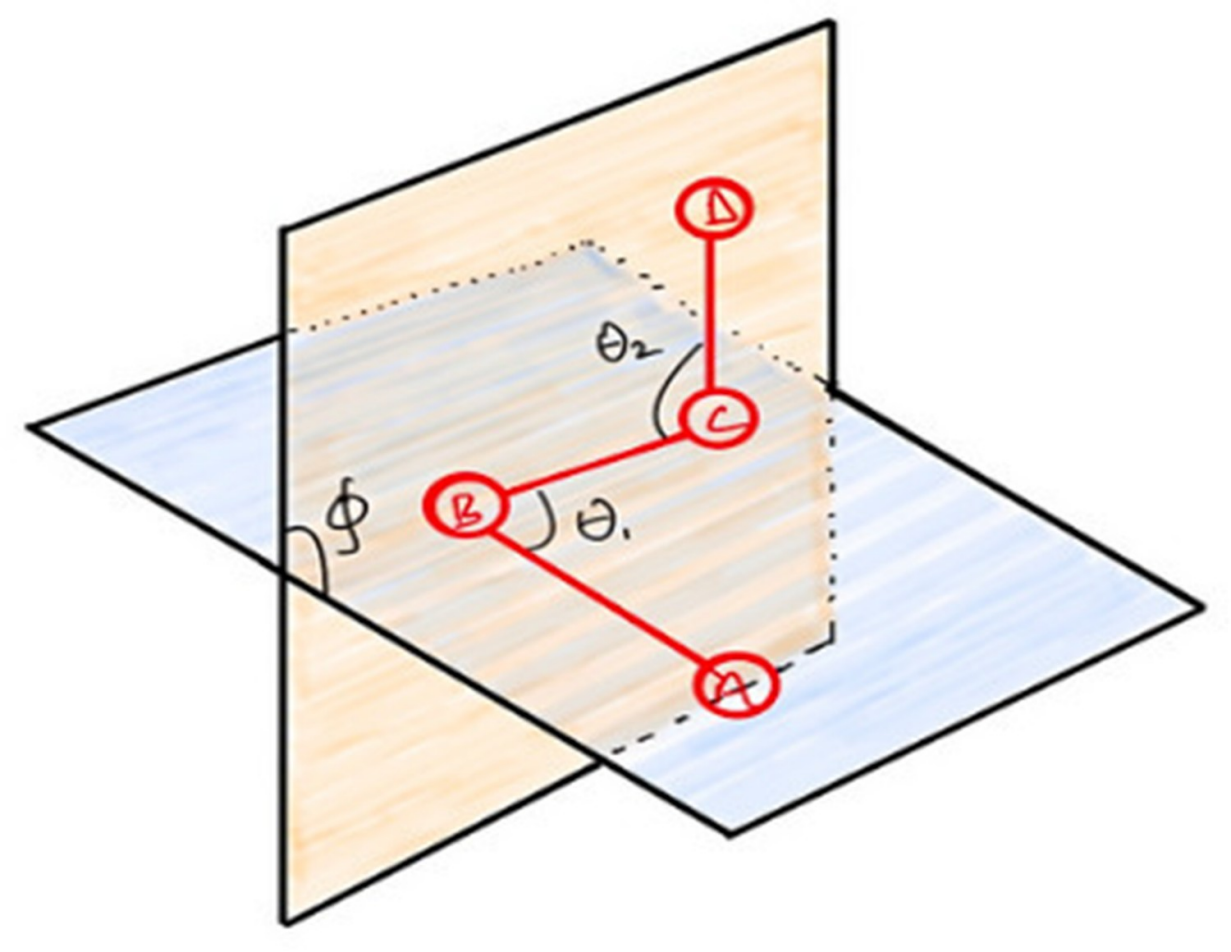
Spatial information for four consecutive atoms.

This section introduces 3D structural information such as bond lengths, bond angles, and the torsion angle used in our new kernel framework. Suppose that the 3D coordinates of two adjacent atoms A and B are given by $\left(x_{A},y_{A},z_{A}\right)$ and $\left(x_{B},y_{B},z_{B}\right)$, respectively. Therefore, the length of the bond between A and B is defined as $d_{AB}=\sqrt{\Delta x^{2}+\Delta y^{2}+\Delta z^{2}}$ where $\Delta x=x_{A}-x_{B},\Delta y=y_{A}-y_{B},\Delta z=z_{A}-z_{B}$. In Fig. 1, the $\theta_{1}$ represents the angle between bond AB and bond BC, which can be obtained by the following expression:


(3) $$\text{cos}\;\theta_{1}=\frac{d_{AB}^{2}+d_{BC}^{2}-d_{AC}^{2}}{2d_{AB}d_{BC}}$$

The torsion angle can also be written in terms of six pairwise distances using the laws of sine and cosine and projections. To define a torsion angle for any molecule, we need at least four consecutive atoms (e.g. A, B, C, and D in Fig. 1), and the plane spanned by atoms A, B, and C and the plane spanned by atoms B, C, and D give the torsion angle ϕ (Fig. 1), where


(4) $$\begin{array}{c} \;\text{cos}(\phi )=\frac{U}{L}\\ U=d_{CD}^{2}\left[1-{\left(\frac{d_{BC}^{2}+d_{CD}^{2}-d_{BD}^{2}}{2d_{BC}d_{CD}}\right)}^{2}\right]\\ +d_{AB}^{2}\left[1-{\left(\frac{d_{AB}^{2}+d_{BC}^{2}-d_{AC}^{2}}{2d_{AB}d_{BC}}\right)}^{2}\right]+d_{AD}^{2}\\ -\frac{1}{4d_{BC}^{2}}{\left(d_{AC}^{2}+d_{BD}^{2}-d_{AB}^{2}-d_{CD}^{2}\right)}^{2}\\ -\frac{1}{\sqrt{2}d_{BC}}d_{AD}\left(d_{AC}^{2}+d_{BD}^{2}-d_{AB}^{2}-d_{CD}^{2}\right)\\ L=2\sqrt{d_{CD}^{2}\left[1-{\left(\frac{d_{BC}^{2}+d_{CD}^{2}-d_{BD}^{2}}{2d_{BC}d_{CD}}\right)}^{2}\right]}\\ \sqrt{d_{AB}^{2}\left[1-{\left(\frac{d_{AB}^{2}+d_{BC}^{2}-d_{AC}^{2}}{2d_{AB}d_{BC}}\right)}^{2}\right]} \end{array}$$

The above Equations (3) and (4) (proof attached in the Supplementary Material) show that for any four consecutive 4 atoms, the six pairwise distances can incorporate 3D structural information such as bond length, bond angle, and torsion angle.

### 3.4 3D adjacency matrix

Here, we define a 3D similarity matrix that gives the bond length information, its square and cube, respectively, give the bond angle and torsion angle information with minor modifications. Let us assume that in graph G the $k^{th}$ atom is A, and there are *T* three-hop pathways starting from it. Let $k_{t}$, say {A-B-C-D}, be the $t^{th}$ path starting from $k^{th}$ atom A. We define a similarity tensor A, such that, the entry $\left(A_{k_{t}}\right)$ of it is a 4×4 matrix contains distance-based similarity of all pairs of atoms from {A-B-C-D}. Here, the entry corresponding to atoms *A* and *B* is defined as ${\left(A_{k_{t}}\right)}_{AB}=\text{exp}(-d_{AB})$ which is bond length similarity between atoms A and B. The adjacency matrix *Adj* is defined for all k and t as:


(5) $$\mathrm{Adj}=\left[\begin{array}{cccc} 0 & 1 & 0 & 0\\ 1 & 0 & 1 & 0\\ 0 & 1 & 0 & 1\\ 0 & 0 & 1 & 0 \end{array}\right].$$

Element-wise multiplication of the simple adjacency matrix *Adj* in Step 4 of Algorithm 1 make sure that $A_{k_{t}}^{1}=A_{k_{t}}⊙\mathrm{Adj}$ captures the distance-based similarity information for four consecutive atoms in a path, while excluding self-loops (diagonal elements) to avoid considering the atom’s self-interaction for bond angle information. The following operation gives us, a similarity measure for the bond angle (ignoring the scaling factor).


(6) $$A_{k_{t}}^{2}={\left(A_{k_{t}}^{1}\right)}^{2}{⊙}_{d}A_{k_{t}},$$

where ${⊙}_{d}$ denotes the element-wise division for nondiagonal entries only. Note that,


(7) $${\left(A_{k_{t}}^{2}\right)}_{AC}=\text{exp}\;\{-(d_{AB}+d_{BC}-d_{AC})\}$$

Similarly, element-wise multiplication and division of matrices ensure that $A_{k_{t}}^{3}$ captures the approximation of torsion angle information for a given set of four nodes.


(8) $$A_{k_{t}}^{3}=(A_{k_{t}}^{2}.A_{k_{t}}^{1}){⊙}_{d}A_{k_{t}}$$

torsion angle similarity captured by the following entry


(9) $$\begin{array}{c} {\left(A_{k_{t}}^{3}\right)}_{AD}=\text{exp}\;\{-(d_{AB}+d_{BC}+d_{CD}-d_{AC}-d_{AD})\}\\ {\left(A_{k_{t}}^{3}\right)}_{DA}=\text{exp}\;\{-(d_{AB}+d_{BC}+d_{CD}-d_{BD}-d_{AD})\} \end{array}$$

Note that, the above expression is one of simple linear approximation for torsion angle in terms of six pairwise distances.

### 3.5 3D Chain Motif Graph Kernel

In this section, we define Chain Motif and novel c-MGK.

Definition 1 (Motif).

*The motif is defined as any connected subgraph arranged in a specific way in 3D space on a given graph* G.

Definition 2 (c-Motif).

*For a given graph* G*, we define the chain motif (c-motif) (denoted as* $v_{c}$*) by any set of four consecutive nodes in* G  *on some graph traversal.*

Definition 3 (c-Motif Graph).

*A c-motif graph is defined by a set of c-motifs as node* $V_{c}=\{v_{c}\}$  *and c-motif edges* $E_{c}$  *and denoted by* $C=\{V_{c},E_{c}\}$*. Here an edge will form between two c-motifs if they share an atom.*

For each $k_{t}^{th}$ c-motif $v_{c}$ ={A-B-C-D} in C, we define the following 3D feature vector:


(10) $$\begin{array}{c} \{{\left(l_{k_{t}}\right)}_{A},{\left(l_{k_{t}}\right)}_{B},{\left(l_{k_{t}}\right)}_{C},{\left(l_{k_{t}}\right)}_{D},{\left(A_{k_{t}}^{1}\right)}_{AB},{\left(A_{k_{t}}^{1}\right)}_{BC},{\left(A_{k_{t}}^{1}\right)}_{CD},\\ {\left(A_{k_{t}}^{1}\right)}_{AC},{\left(A_{k_{t}}^{1}\right)}_{BD},{\left(A_{k_{t}}^{1}\right)}_{AD},{\left(A_{k_{t}}^{2}\right)}_{AC},\\ {\left(A_{k_{t}}^{2}\right)}_{BD},{\left(A_{k_{t}}^{3}\right)}_{AD}+{\left(A_{k_{t}}^{3}\right)}_{DA}\} \end{array}$$

The first four are the labels of the nodes in the c-motif; the next six are the bond length; the next two are the bond angle; and the last one contains the torsion angle information. We now have a graph with continuous 3D attributes, and The GHK (Feragen *et al.* 2013) provides a way to measure the similarity between graphs, even when those graphs have continuous, multi-dimensional node attributes. Hence we define the 3D c-motif graph kernel by defining the GHK on top of the c-motif graph, as demonstrated below.


(11) $$\begin{array}{c} K(G,G^{\prime })=K(C,C^{\prime })\\ =\sum_{v\in V_{c}}\sum_{v^{\prime }\in V_{c}^{\prime }}w(v,v^{\prime })k_{n_{c}}(v,v^{\prime }) \end{array}$$

Where $w(v,v^{\prime })$ is the same weight as defined in GHK Equation (2). It counts the number of times *v* and $v^{\prime }$ appear at the same hop and $k_{n_{c}}(v,v^{\prime })$ is the multiplication of Dirac delta kernel on sets of node and linear kernel of continuous node attributes defined in Equation (10). Applying the GHK on top of the c-motif graph will result in high similarity, i.e. a sequence of c-motifs that are present in both graphs.

Algorithm 1. 3-dimensional feature for a $k_{t}^{th}$ path

**Require**: Path {A-B-C-D} and 3-dimensional information for G.

**Ensure**: 3-dimensional feature.

 1: Adj← adjacency matrix for consecutive nodes [Equation (5)].

 2: $d_{k_{t}}\leftarrow$ create 4×4 distance matrix for {A-B-C-D}

 3: Update $A_{k_{t}}\leftarrow \;\text{exp}(-d_{k_{t}})$

 4: Update $A_{k_{t}}^{1}\leftarrow A_{k_{t}}⊙\mathrm{Adj}$

 5: Update $A_{k_{t}}^{2}\leftarrow {\left(A_{k_{t}}^{1}\right)}^{2}{⊙}_{d}A_{k_{t}}$

 6: Update $A_{k_{t}}^{3}\leftarrow (A_{k_{t}}^{2}.A_{k_{t}}^{1})$${⊙}_{d}$$A_{k_{t}}$

 7: Compute bond length, bond angle, and torsion angle similarity using $A_{k_{t}}^{1},A_{k_{t}}^{2}$ and $A_{k_{t}}^{3}$, respectively and store in a single vector.

 8: **return** 3-dimensional feature vector.

Algorithm 2. 3-dimensional c-motif graph formation

**Require**: Paths and 3-dimensional information for G.

**Ensure**: c-motif graph C.

1: HFV = []

2: Initialize Graph C.

3: **for** each path $k_{t}$ in Paths **do**

4:   Compute 3-dimensional feature using Algorithm 1 and store in HFV.

5: **end for**

6: There is an edge between two c-motif if there is a common atom in two c-motif.

7: $C(V_{c},E_{c})\leftarrow$ c-motif graph with node set $V_{c}$ and edge set $E_{c}$.

8: **return**  $C(V_{c},E_{c})$

Theorem 1. *The complexity of the 3-dimensional c-motif graph kernel (c-MGK) for two graphs is* $O(n^{2}T^{6}(nT^{4}$  $+\text{log}(nT^{3})+\delta^{2}+d))$, *where n is the maximum number of nodes in a graph, T is the highest degree of a node* , δ  *is the diameter of the graph, and d are the dimensions of node attributes.*

Proof.

Three-hop paths for a given graph have a time complexity of $O(nT^{3})$ and to construct the c-motif graph Algorithm 2 has a time complexity of $O(n^{2}T^{6})$. The complexity of the GHK with graph size *n* is $O\left(n^{2}(m+\text{log}(n)+\delta^{2}+d))\right)$ (Feragen *et al.* 2013). Therefore, the overall complexity for the computation of the kernel between two graphs of size $nT^{3}$ is $O(2nT^{3}+2n^{2}T^{3}+n^{2}T^{6}(nT^{4}+\text{log}(nT^{3})+\delta^{2}+d))$. In the c-motif graph, every node has *T* possible choices for edges, and this increases the number of edges to $nT^{4}$. ▪

### 3.6 Efficient 3DGHK

This section proposes an efficient version of the c-motif graph kernel that uses the 3D feature vector of the nodes but reduces the computational complexity associated with calculating the kernel for larger graphs, especially those with high-degree nodes. The challenge we are addressing is the exponential growth of the number of three-hop neighbors, which can lead to impractical computation times for GHK on c-motif graphs of large graphs. To tackle this, we are introducing the 3DGHK, which maintains the ability to compute similarities between graphs with continuous node attributes (bond lengths, bond angles, torsion angles) while reducing computational complexity.

We define a 3D node kernel on the original graph instead of defining a kernel on the c-motif.


(12) $$\begin{array}{c} K(G,G^{\prime })=\sum_{v\in V}\sum_{v^{\prime }\in V^{\prime }}w(v,v^{\prime })k_{n_{3D}}(v,v^{\prime }) \end{array}$$

Where $w(v,v^{\prime })$ is the same weight as defined in GHK Equation (2).


(13) $$k_{n_{3D}}(v,v^{\prime })=k_{n}(v,v^{\prime })k_{\mathrm{neigh}}(v,v^{\prime })$$

where , $k_{n}(,)$ is a the Dirac delta (0 or 1) node kernel that finds similarities between the two current nodes and $k_{\mathrm{neigh}}(,)$ is a 3-hop neighborhood kernel that first discovers the all common c-motifs starting from nodes *v* and $v^{\prime }$ by comparing labels, then take a sum over the 3D similarity between the common c-motifs using nine features $\{{\left(A_{k_{t}}\right)}_{AB},$  ${\left(A_{k_{t}}\right)}_{BC},{\left(A_{k_{t}}\right)}_{CD},{\left(A_{k_{t}}\right)}_{AC},{\left(A_{k_{t}}\right)}_{BD},{\left(A_{k_{t}}\right)}_{AD},{\left(A_{k_{t}}^{2}\right)}_{AC},{\left(A_{k_{t}}^{2}\right)}_{BD},{\left(A_{k_{t}}^{3}\right)}_{AD}+{\left(A_{k_{t}}^{3}\right)}_{DA}\}$


$$k_{\mathrm{neight}}(v,v^{\prime })=\sum_{p_{v}\in p_{3}(v)}\sum_{p_{v^{\prime }}\in p_{3}(v^{\prime })}\Delta_{p_{v}=p_{v^{\prime }}}k_{3hop}(p_{v},p_{v^{\prime }})$$

Where $p_{3}(v)$ denotes set of 3-hop neighbors of a node v∈G and Δ equal to 1 if paths are $p_{v}$ and $p_{v^{\prime }}$ have same atoms, otherwise 0. For our experiment, we used the $k_{3hop}(\cdot ,\cdot )$ kernel as a linear kernel.

Theorem 2. *The time complexity for the 3-dimensional graph hop kernel between two graphs is*  $O(n^{2}(m+\text{log}(n)+\delta^{2}+dT^{6}))$  *where n is the maximum number of nodes in a graph, T is the highest degree of a node, m is the number of edges*, δ  *is the diameter of the graph, and d is the dimensions of node attributes.*

Proof.

If we know $w(v,v^{\prime })$ and $k_{n_{3D}}$, then the kernel $K(G,G^{\prime })$ can be computed in $O(n^{2})$ time. It is demonstrated by Feragen *et al.* (2013) that the computation time for $w(v,v^{\prime })$ for all v∈V and $v^{\prime }\in V^{\prime }$ is $O(2n^{2}\delta^{2}+n^{2}(m+\text{log}(n)+\delta^{2}))$. Our 3-dimensional node kernel is a combination of a node kernel and a neighborhood structural kernel. Any node kernel for all v∈V and $v^{\prime }\in V^{\prime }$ can be computed in $O(n^{2}d)$ time (Feragen *et al.* 2013), where d denotes the dimension of the feature vector. To find the neighborhood structural similarity $K_{\mathrm{nbd}}(v,v^{\prime })$ between two nodes, we need to compare all possible three-hop neighbors structural similarity; therefore, it takes $O(T^{3}T^{3}d)$ time. For all v∈V and $v^{\prime }\in V^{\prime }$, the neighborhood structural kernel can be computed in $O(n^{2}T^{6}d)$ time. It follows that the time complexity for computing $K(G,G^{\prime })$ is $O(4nT^{3}+n^{2}+2n^{2}\delta^{2}+n^{2}(m+\text{log}(n)+\delta^{2})+n^{2}d+n^{2}T^{6}d)$  $\approx O(n^{2}(m+\text{log}(n)+\delta^{2}+dT^{6})).$ ▪

Algorithm 3. 3-dimensional attributes for each nodes

**Require**: Paths and 3-dimensional information for G.

**Ensure**: 3-dimensional Feature.

1: FV = {}

2: **for** each node *k* in G  **do**

3:    FVP = {}

4:    **for** each 3-hop path *t* of node *k*  **do**

5:      Compute the 3-dimensional feature for path $k_{t}$ using Algorithm 1 and store atom type as key and rest as value in FVP.

6:    **end for**

7:    Store FVP in FV where key is node and value is a dictionary FVP

8: **end for**

9: **return** FV.

#### 3.6.1 Translation and rotational invariance

The proposed kernels depend on the feature vectors defined in Equation (10) and those features depend on the Euclidean distance among atoms belonging to the three-hop path. Euclidean distance is invariant to translation and rotation hence the proposed kernels are also translational and rotational invariant.

## 4 Experiments and results

In this section, we (1) compare the performance of the proposed efficient 3DGHK works in the molecule classification task with other benchmarks, (2) study the runtime of the proposed 3DGHK against other 2D and 3D kernels and deep learning models, and (3) carried out an ablation study to show the importance of the 3D features taken in the proposed kernel.

### 4.1 Dataset

For the evaluation of our kernel, we have used two key sources. The first group includes nine datasets, each containing lists of known active and inactive compounds across various target protein classes, as detailed in Liu *et al.* (2023). The second collection is the tox21 datasets (Huang *et al.* 2016, Mayr *et al.* 2016) available as open source. The Tox21 dataset includes information on 12 707 chemical molecules and their activity across 12 toxicological experiments. The dataset has been processed, with molecules converted from SMILES to the 3D SDF format using OpenBabel (O’Boyle *et al.* 2011), following the procedure outlined in Liu *et al.* (2023). The statistics for these two datasets are attached to the Supplementary Materials.

### 4.2 Benchmark

We are benchmarking our graph kernels (c-MGK and 3DGHK) with five other graph kernels, i.e. PK (Mahé *et al.* 2006), GHK (Feragen *et al.* 2013), RWK (Gärtner *et al.* 2003), SPK (Borgwardt and Kriegel 2005), WLK (Shervashidze *et al.* 2011), and two deep learning models based on graph convolutional network (GCN) (Kipf and Welling 2016) and GCN-Attr. The PK is the 3D graph kernel defined for the virtual screening task. The others are the state-of-the-art 2D kernels currently used for graph classification.

### 4.3 Experimental details

The dataset we are working is highly unbalanced (as mentioned in the Supplementary Tables S1 and S2). There are far more inactive molecules than active ones, which might skew the results if not addressed. To tackle this, we randomly select an equal number of inactive molecules as active molecules. This creates a balanced dataset, ensuring that both classes (active and inactive) are represented equally. The same data have been used for all benchmarking models. We divided this dataset into 70% 15% 15% for training, validation, and testing. Our goal is to compare the performance of the proposed c-MGK and 3DGHK with other 2D, 3D kernels, and deep learning models to see if the addition of 3D structural information makes the overall performance better for the molecular classification task. For all kernels, we have used the SVM classifier with hyperparameter C tuned by grid search in the range {0.001,0.01,0.1,1,10,100,1000}. We implemented the PK Mahé *et al.* (2006) ourselves, while for other kernels (Gärtner *et al.* 2003, Borgwardt and Kriegel 2005, Shervashidze *et al.* 2011, Feragen *et al.* 2013), we used the Grakel library (Siglidis *et al.* 2018). For the deep learning model, we implemented the GCN architecture as described in Zhang *et al.* (2019) and Baek *et al.* (2021), with 4(GCN)and 3(GCN-Attr) hidden layers and mean pooling with hidden units selected from {16,32,64} by validation accuracy. We trained the model for 100 epochs with the Adam (Kingma 2014) optimizer with learning rate and dropout tuned by grid search among {0.1,0.01,0.001} and {0.2, 0.4, 0.7}. For all experiments, we used the node label, adjacency matrix, and 3D coordinate (if applicable) information. But one can easily incorporate node attributes and edge attributes by merging them into a feature vector for each c-motif. GCN-Attr used other features described in Zhang *et al.* (2019) and Baek *et al.* (2021).

### 4.4 Performance on classification

Our experiments in Tables 1 and 2 show that the proposed 3DGHK outperforms the current state-of-the-art graph kernels. Out of 21 datasets, for nine datasets, 3DGHK has the best accuracy, and for nine datasets, 3DGHK has the second-best accuracy. For almost every dataset except one, 3DGHK is giving better results than all state-of-the-art 2DGHKs. Using 3DGHK, we could achieve an improvement of as much as 6% over the state-of-the-art. This shows that incorporating 3D structural information actually helps.

**Table 1.** Comparisons between different graph kernels for dataset in Liu *et al.* (2023).^a^

Dataset	3DGHK	c-MGK	PK	GHK	RWK	SPK	WLK	GCN	GCN-Attr	↑ **acc.**
1798	0.61±0.07	**0.64** ±**0.06**	0.53± 0.05	0.52± 0.06	0.52 ± 0.04	0.59± 0.06	0.59 ± 0.07	0.50 ±0.03	0.53 ±0.05	≈5%
1843	**0.77** ± **0.06**	0.69±0.04	0.61± 0.05	0.67± 0.04	0.62±0.04	0.70± 0.05	0.72± 0.06	0.62 ± 0.08	0.75 ±0.06	≈2%
2258	**0.71** ± **0.04**	0.66± 0.05	0.53± 0.05	0.59± 0.06	0.58 ± 0.05	0.63±0.07	0.70±0.04	0.60 ±0.06	0.69 ±0.05	≈1%
2689	0.75±0.06	**0.75** ± **0.06**	0.61± 0.05	0.62± 0.05	0.61±0.07	0.59± 0.08	0.73 ±0.06	0.59 ±0.06	**0.80** ±**0.04**	–
435008	0.60± 0.06	0.63±0.05	0.58± 0.04	0.52± 0.06	0.55 ± 0.06	0.63 ± 0.06	0.67 ± 0.046	0.58 ± 0.06	**0.68** ±**0.0**	–
435034	0.68 ±0.04	0.67 ±0.04	>2d	0.58 ± 0.04	0.60± 0.03	0.67 ±0.04	**0.69** ±**0.04**	0.65 ± 0.05	0.68 ±0.04	–
463087	0.73 ±0.03	0.69 ±0.03	>2d	0.63 ± 0.04	0.62± 0.03	0.70 ±0.04	**0.74** ±**0.05**	0.49 ± 0.01	0.72 ±0.03	–
485290	0.69 ± 0.06	0.70 ± 0.05	>2d	0.60± 0.05	0.62± 0.04	0.70 ± 0.071	0.71 ± 0.05	0.66 ± 0.05	**0.73** ±**0.04**	–
488997	**0.70** ± **0.06**	0.64 ± 0.06	>2d	0.55 ±0.05	0.56±0.05	0.62 ± 0.06	0.61 ±0.05	0.60 ± 0.06	0.64 ±0.05	≈6%

a After 20 runs, we reported the mean accuracy with the SD. Blue and cyan indicate the highest and the second highest accuracy in a row, respectively.

**Table 2.** Comparisons between different graph kernels for tox21 dataset (Mayr *et al.* 2016).^a^

Dataset	3DGHK	c-MGK	PK	GHK	RWK	SPK	WLK	GCN	GCN-Attr	↑ **acc.**
AhR	0.78 ± 0.02	0.72 ± 0.02	0.63 ± 0.03	0.65 ± 0.02	0.63 ± 0.03	0.67 ± 0.03	0.77 ± 0.02	0.51 ± 0.012	**0.80** ± **0.03**	–
AR	0.74 ±0.04	0.68 ±0.04	0.74 ± 0.04	**0.75** ± **0.03**	0.74 ± 0.03	0.74 ± 0.03	0.73 ± 0.03	0.60 ± 0.05	**0.75** ± **0.03**	–
AR.LBD	0.77±0.04	0.69 ±0.05	0.76 ± 0.04	0.76± 0.05	0.76 ± 0.05	0.78 ±0.05	0.77 ± 0.03	0.58± 0.06	**0.79** ± **0.04**	–
Aromatase	**0.73** ± **0.04**	0.63 ±0.04	0.67 ± 0.04	0.67 ± 0.03	0.69 ±0.03	0.70 ±0.03	**0.73** ±**0.04**	0.50 ±0.026	0.69 ± 0.04	–
ER	0.69 ± 0.02	0.63 ±0.03	0.68 ± 0.02	0.62 ± 0.02	0.62±0.03	0.67 ± 0.02	**0.70** ± **0.02**	0.53 ± 0.03	0.67 ± 0.02	–
ER.LBD	0.72 ± 0.04	0.67 ±0.04	0.68 ± 0.04	0.65 ± 0.04	0.65 ± 0.03	0.70± 0.03	0.72 ± 0.04	0.51 ± 0.03	**0.73** ± **0.03**	–
PPAR.g	**0.71** ± **0.05**	0.60 ±0.05	0.62± 0.05	0.62 ± 0.05	0.63 ± 0.04	0.63 ± 0.04	0.64 0.04	0.53 ± 0.04	0.65 ± 0.06	≈6%
ARE	**0.70** ±**0.02**	0.60 ±0.02	0.60 ± 0.03	0.64 ± 0.02	0.64 ± 0.03	0.62 ± 0.03	0.68 ± 0.02	0.51 ± 0.020	0.68 ± 0.02	≈2%
ATAD5	**0.69** ± **0.04**	0.62 ±0.06	0.58 ± 0.05	0.62 ± 0.04	0.59 ± 0.05	0.60 ± 0.05	**0.69** ± **0.04**	0.50 ± 0.01	0.68 ± 0.05	–
HSE	**0.66** ± **0.04**	0.60 ±0.03	0.56 ± 0.04	0.59±0.04	0.59±0.04	0.60±0.04	0.65±0.04	0.56±0.040	0.62 ± 0.05	≈1%
MMP	**0.78** ± **0.03**	0.69 ±0.03	0.67± 0.02	0.68± 0.03	0.65 ± 0.02	0.68 ± 0.02	0.78 ± 0.02	0.51 ± 0.014	**0.80** ± **0.02**	–
p53	**0.76** ± **0.03**	0.63 ±0.04	0.60 ± 0.04	0.68 ± 0.02	0.66 ± 0.03	0.65 ± 0.03	0.70 ± 0.03	0.54 ± 0.02	0.71 ± 0.03	≈5%

a After 20 runs, we reported the mean accuracy with the SD. Blue and cyan indicate the highest and second highest accuracy for a row, respectively.

**Figure 2.**
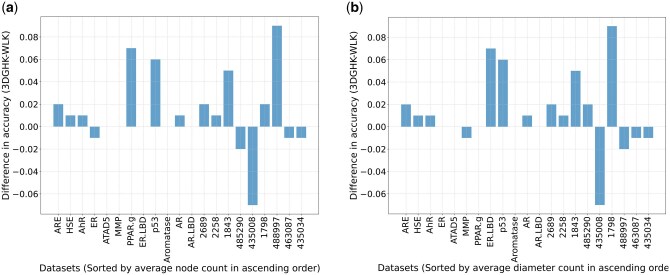
Difference in performance (3DGHK–WLK) according to molecular size, i.e. average number of nodes (left), average diameter length (right).

Moreover, the proposed method outperforms the state-of-the-art 3D PK on all datasets. In practice, for many datasets, the PK computation took more than 2 days. In those cases, we were unable to complete those experiments, which are indicated in the tables as “>2d”. Although the WLK outperforms the 3DGHK on five datasets, 3DGHK outperforms WLK on 11 datasets and achieves comparable performance on five others. Notably, 3DGHK demonstrates superior performance compared to the WL kernel. Datasets with IDs 488997, PPAR.g, and p53 show the most significant improvements, with gains of ∼9%, 7%, and 6%, respectively, over the WL kernel. These results emphasize the value of incorporating 3D data into graph-based analyses. For continuous attributed graphs, our kernel 3DGHK is applicable while the WL kernel is defined only for discrete node attributes. We also observed (Fig. 2) that 3DGHK captures local structural information more accurately for smaller (average node, average length of diameter) graphs. After excluding the five datasets with larger graphs based on average node and average diameter, we performed statistical tests resulting in the values 0.04322 and 0.00705, respectively. The *P*-values from these tests demonstrate that 3DGHK significantly outperforms WLK in datasets consisting of smaller molecular graphs.

We also compare 3DGHK with GCN-Attr, i.e. GCN with extended features along with the node label as the initial attribute. This represents a pessimistic comparison of our model, as it uses only the node label as a feature. Nevertheless, we found that the performance of 3DGHK is on par with that of GCN-Attr. For a few datasets, 3DGHK even outperforms GCN-Attr slightly. Since other models do not use extended features, we also fairly compare the proposed model with GCN, where only the node label is used as the initial feature. In this comparison, 3DGHK performs significantly better.

From Tables 1 and 2, we observe that the c-MGK performs as well as or better than other kernels across the datasets. Specifically, c-MGK achieves the highest accuracy in two datasets and the second-best accuracy in 5 of the 21 datasets. Our findings indicate that c-MGK performs better on Data 1 (Liu *et al.* 2023) (Supplementary Table S1) compared to Data 2 (Huang *et al.* 2016; Mayr *et al.* 2016) (Supplementary Table S2). This difference can be attributed to the average number of nodes in the graphs: Data 1 contains graphs with significantly more nodes than Data 2. For example, in the case of p53 protein graphs within Data 2, 147 graphs have fewer than 10 nodes each. In such small graphs, very few nodes have neighbors that are three hops away, which reduces the availability of 3D structural information. When this occurs, the weight vector $w(v,v^{\prime })$ plays a critical role in influencing the kernel values. The matrices used to compute $w(v,v^{\prime })$ in the graph hopper framework (Feragen *et al.* 2013) differ due to discrepancies between the original graph and the c-motif graph. The c-motif graph construction process accounts for these differences, which explains the varying performance of c-MGK and 3DGHK. Consequently, the reduced availability of 3D information in small graphs impacts the relative accuracy of these kernels.

The *P*-value in Table 3 shows the statistically significant difference between our 3DGHK and 2D graph kernels and the deep learning model.

**Table 3.** Reported *P*-values (upto five decimal places) between 3DGHK and PK, GHK, RWK, SPK, WLK, GCN, GCN-Attr.

	3DGHK		3DGHK
**PK**	0.00002	**GHK**	0.00000
**RWK**	0.00009	**SPK**	0.00051
**WLK**	0.08587	**GCN**	0.00000
**GCN-Attr**	0.37902		

### 4.5 Hypothesis testing

To know if the results in Tables 1 and 2 are statistically significant, we perform hypothesis testing for them. We conducted a pairwise Wilcoxon signed rank test between our kernel and other kernels, displaying the *P*-values up to five decimal places in Table 3. It shows results in Tables 1 and 2 are statistically significant, except the WL kernel and GCN-Attr with extended features.

### 4.6 Runtime comparison

**Figure 3.**
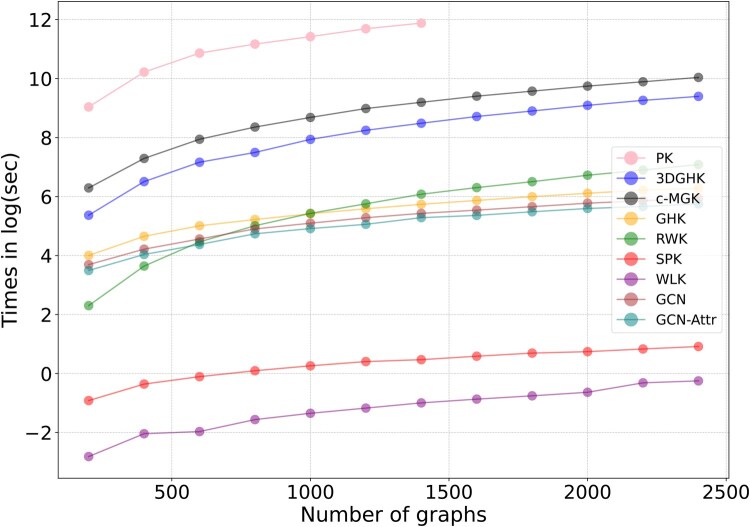
Runtime in $log_{e}$ of seconds to train a model.

An empirical evaluation of runtime was conducted to assess model training time relative to the number of graphs *N*, selected from the range [200,400,…,2400] and sampled from the ARE-tox21 dataset. Figure 3 plots the log_e_ of training time against the number of graphs.

**Figure 4.**
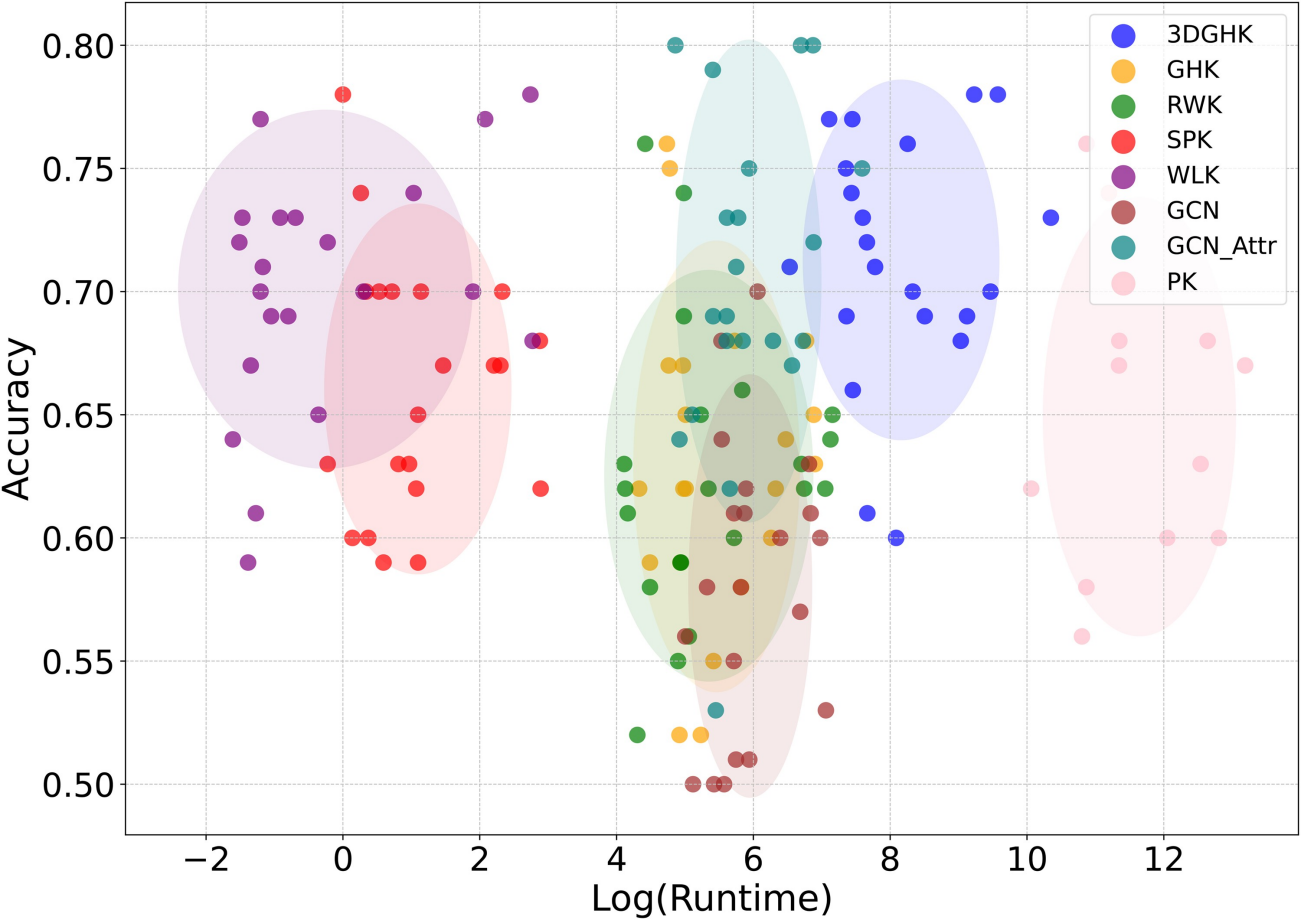
Scatter plots for runtime versus accuracy with superimposed shaded ellipse for each models.

Results indicate that the proposed 3DGHK is slower than 2D kernels and GCN-based methods but faster than the 3D PK, which required over 2 days to process a large number of graphs and hence was only tested up to 1400 graphs. Additionally, the figure highlights that the 3D c-MGK exhibits exponentially slower runtime compared to 3DGHK. Figure 4 shows the tradeoff between accuracy and runtime. The ellipses represent the variance in a model’s performance across all datasets. Although GCN-Attr performs better in some cases, its variance is higher than that of the proposed 3DGHK. The performance of 3DGHK, in terms of accuracy, is superior to other baselines but comes at the cost of increased runtime.

In a nutshell, 3DGHK can consider the 3D structural information efficiently. We have also observed that it captures structural similarity accurately when the molecule has a short diameter. On the other hand, the kernel computation is high for larger graphs and higher than 2D kernels.

### 4.7 Ablation study

An ablation study was conducted to evaluate the significance of including 3-hop neighbors in the proposed 3DGHK. The study replaced the structural similarity feature of the 3-hop path with that of the 2-hop path. The framework, which originally utilizes four node labels, six pairwise distances, two bond angles, and one torsion angle, was simplified for the 2-hop kernel to include three node labels, three pairwise distances, and one bond angle. Results, as shown in Tables 4 and 5, indicate a drop in accuracy with reduced information, highlighting the critical role of 3-hop neighborhood data. While extending to neighbors beyond 3-hop could capture more information, it would significantly increase time complexity due to the larger size of the adjacency matrix required for structural feature extraction. For the ablation study, we have used an SVM classifier with fixed *C* = 1.

**Table 4.** Comparison based on 3-hop neighbors versus 2-hop neighbors in our framework.

Dataset	With 3-hops	With 2-hops
1798	0.61±0.044	0.55 ±0.061
1843	0.75±0.065	0.67±0.062
2258	0.71± 0.045	0.59±0.046
2689	0.73±0.049	0.57±0.057
435008	0.58± 0.058	0.50±0.056
435034	0.64 ±0.036	0.60±0.035
463087	0.69 ±0.030	0.63±0.037
485290	0.67 ± 0.053	0.57±0.065
488997	0.62±0.049	0.55±0.071

**Table 5.** Comparison based on 3-hop neighbors versus 2-hop neighbors in our framework.

Dataset	With 3-hops	With 2-hops
AhR	0.71 ± 0.024	0.65±0.028
AR	0.72 ±0.045	0.72± 0.037
AR.LBD	0.74±0.036	0.74±0.046
Aromatase	0.70 ± 0.026	0.67 ±0.026
ER	0.65 ± 0.02	0.63 ±0.026
ER.LBD	0.69 ± 0.032	0.68±0.033
PPAR.g	0.61 ± 0.042	0.61±0.053
ARE	0.65 ±0.021	0.63±0.028
ATAD5	0.63 ± 0.037	0.61 ±0.038
HSE	0.63±0.030	0.59±0.039
MMP	0.72±0.018	0.66±0.016
p53	0.67 ± 0.025	0.64 ± 0.030

## 5 Conclusion

This work introduces two novel 3D graph kernels for virtual screening: the 3D c-MGK and the 3DGHK. These kernels leverage 3D structural information, including bond lengths, angles, and torsion angles of molecules, within a graph kernel framework. The kernels were evaluated against state-of-the-art 2D and 3D kernels for ligand-based virtual screening. Theoretical and empirical analyses of time complexity, ablation studies on three-hop neighbor paths, and hypothesis testing underscore the importance of incorporating 3D structural data. Results show that using 3D information enhances model performance, albeit with added computational cost.

## Supplementary Material

btaf208_Supplementary_Data
