# Supplementary material for: Efficient 3D kernels for molecular property prediction
Source: Bioinformatics. 2025 Jul 15;41(Suppl 1):i58–67. doi: 10.1093/bioinformatics/btaf208 (PMC12261455; doi:10.1093/bioinformatics/btaf208)
Supplement: btaf208_Supplementary_Data [file btaf208_supplementary_data.zip › btaf208_Supplementary_Data/Ankit.49.alternative_text.pdf]

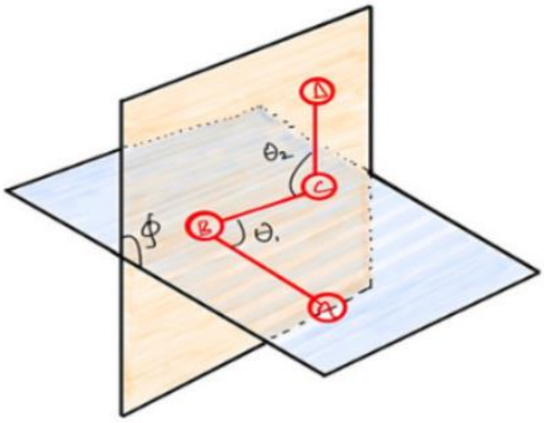

FIGURE 1. SPATIAL INFORMATION FOR FOUR CONSECUTIVE ATOMS.

Alt text: Four consecutive atoms in 3D space showing two bond angles and one torsion angle.

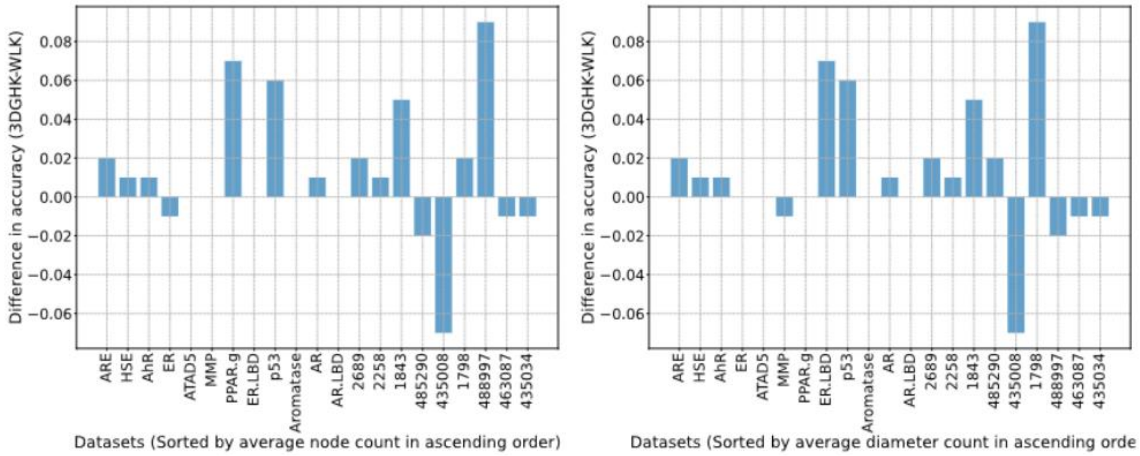

FIGURE 2. DIFFERENCE IN PERFORMANCE (3DGHK - WLK) ACCORDING TO MOLECULAR SIZE, I.E., AVERAGE NUMBER OF NODES (LEFT), AVERAGE DIAMETER LENGTH (RIGHT).

Alt Text: Two bar plots. The left one shows the better performance of 3DGHK for molecules with a lower number of nodes. The right one shows the better performance of 3DGHK for molecules with a smaller diameter.

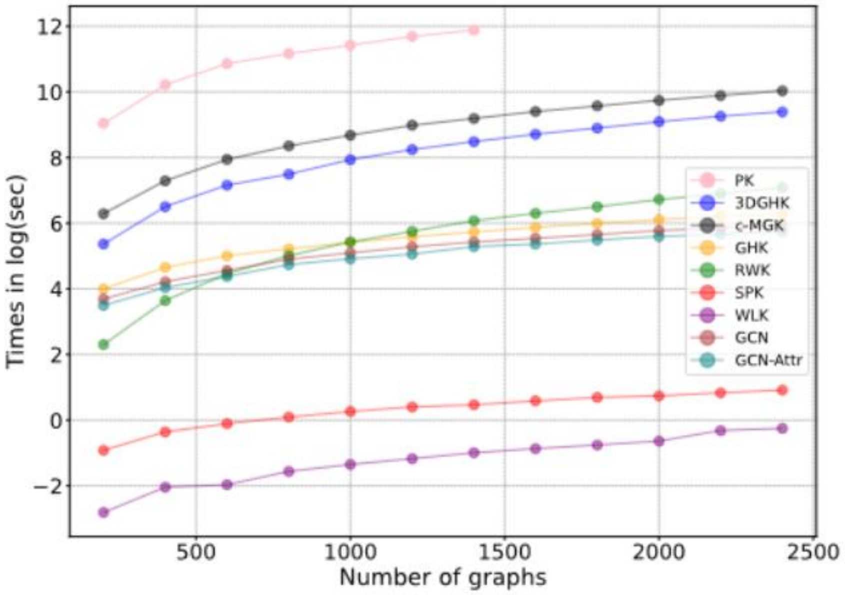

FIGURE 3. RUNTIME IN LOGE OF SECONDS TO TRAIN A MODEL.

Alt text. A line plot compares the runtime of different models as the graph size increases. The two proposed 3D kernels are faster than their 3D counterpart, PK, but slower than the remaining 2D kernels and deep learning model.

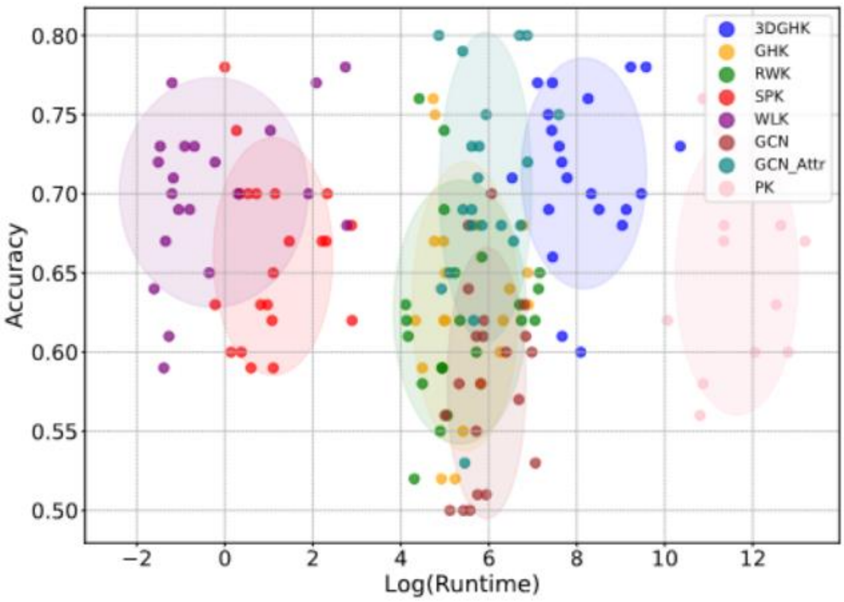

FIGURE 4. SCATTER PLOTS FOR RUNTIME VS ACCURACY WITH SUPERIMPOSED SHADDED ELLIPSE FOR EACH MODELS.

Alt text: A scatter plot between accuracy and log(runtime) for different baselines across different datasets. Each is represented by the different colours, and the shaded ellipses of the same colour are superimposed to show the underlined distribution.

TABLE 1. COMPARISONS BETWEEN DIFFERENT GRAPH KERNELS FOR DATASET . AFTER 20 RUNS, WE REPORTED THE MEAN ACCURACY WITH THE STANDARD DEVIATION. BLUE AND CYAN INDICATE THE HIGHEST AND THE SECOND HIGHEST ACCURACY.

Alt text: Table 2 summarises the predictive accuracy with a standard deviation for the different baseline models evaluated for dataset 2.

Dataset	3DGHK	c-MGK	PK	GHK	RWK	SPK	WLK	GCN	GCN-Attr	↑ acc.
1798	0.61±0.07	**0.64 ±0.06**	0.53± 0.05	0.52± 0.06	0.52 ± 0.04	0.59± 0.06	0.59 ± 0.07	0.50 ±0.03	0.53 ±0.05	≈ 5%
1843	**0.77±0.06**	0.69±0.04	0.61± 0.05	0.67± 0.04	0.62±0.04	0.70± 0.05	0.72± 0.06	0.62 ± 0.08	0.75 ±0.06	≈ 2%
2258	**0.71± 0.04**	0.66± 0.05	0.53± 0.05	0.59± 0.06	0.58 ± 0.05	0.63±0.07	0.70±0.04	0.60 ±0.06	0.69 ±0.05	≈ 1%
2689	0.75±0.06	**0.75±0.06**	0.61± 0.05	0.62± 0.05	0.61±0.07	0.59± 0.08	0.73 ±0.06	0.59 ±0.06	**0.80 ±0.04**	-
435008	0.60± 0.06	0.63±0.05	0.58± 0.04	0.52± 0.06	0.55 ± 0.06	0.63 ± 0.06	0.67 ± 0.046	0.58 ± 0.06	**0.68 ±0.0**	-
435034	0.68 ±0.04	0.67 ±0.04	>2d	0.58 ± 0.04	0.60± 0.03	0.67 ±0.04	**0.69 ±0.04**	0.65 ± 0.05	0.68 ±0.04	-
463087	0.73 ±0.03	0.69 ±0.03	>2d	0.63 ± 0.04	0.62± 0.03	0.70 ±0.04	**0.74 ±0.05**	0.49 ± 0.01	0.72 ±0.03	-
485290	0.69 ± 0.06	0.70 ± 0.05	>2d	0.60± 0.05	0.62± 0.04	0.70 ± 0.071	0.71 ± 0.05	0.66 ± 0.05	**0.73 ±0.04**	-
488997	**0.70±0.06**	0.64 ± 0.06	>2d	0.55 ±0.05	0.56±0.05	0.62 ± 0.06	0.61 ±0.05	0.60 ± 0.06	0.64 ±0.05	≈ 6%

TABLE 2 . COMPARISONS BETWEEN DIFFERENT GRAPH KERNELS FOR TOX21 DATASET . AFTER 20 RUNS, WE REPORTED THE MEAN ACCURACY WITH THE STANDARD DEVIATION. BLUE AND CYAN INDICATE THE HIGHEST AND SECOND HIGHEST ACCURACY.

Alt text: Table 2 summarises the predictive accuracy with a standard deviation for the different baseline models evaluated for dataset 2.

Dataset	3DGHK	c-MGK	PK	GHK	RWK	SPK	WLK	GCN	GCN-Attr	↑ acc.
AhR	0.78 ± 0.02	0.72 ± 0.02	0.63 ± 0.03	0.65 ± 0.02	0.63 ± 0.03	0.67 ± 0.03	0.77 ± 0.02	0.51 ± 0.012	**0.80 ± 0.03**	-
AR	0.74 ±0.04	0.68 ±0.04	0.74 ± 0.04	**0.75 ± 0.03**	0.74 ± 0.03	0.74 ± 0.03	0.73 ± 0.03	0.60 ± 0.05	**0.75 ± 0.03**	-
AR.LBD	0.77±0.04	0.69 ±0.05	0.76 ± 0.04	0.76± 0.05	0.76 ± 0.05	0.78 ±0.05	0.77 ± 0.03	0.58± 0.06	**0.79 ± 0.04**	-
Aromatase	**0.73 ± 0.04**	0.63 ±0.04	0.67 ± 0.04	0.67 ± 0.03	0.69 ±0.03	0.70 ±0.03	**0.73 ±0.04**	0.50 ±0.026	0.69 ± 0.04	-
ER	0.69 ± 0.02	0.63 ±0.03	0.68 ± 0.02	0.62 ± 0.02	0.62±0.03	0.67 ± 0.02	**0.70 ± 0.02**	0.53 ± 0.03	0.67 ± 0.02	-
ER.LBD	0.72 ± 0.04	0.67 ±0.04	0.68 ± 0.04	0.65 ± 0.04	0.65 ± 0.03	0.70± 0.03	0.72 ± 0.04	0.51 ± 0.03	**0.73 ± 0.03**	-
PPAR.g	**0.71 ± 0.05**	0.60 ±0.05	0.62± 0.05	0.62 ± 0.05	0.63 ± 0.04	0.63 ± 0.04	0.64 ± 0.04	0.53 ± 0.04	0.65 ± 0.06	≈ 6%
ARE	**0.70 ±0.02**	0.60 ±0.02	0.60 ± 0.03	0.64 ± 0.02	0.64 ± 0.03	0.62 ± 0.03	0.68 ± 0.02	0.51 ± 0.020	0.68 ± 0.02	≈ 2%
ATAD5	**0.69 ± 0.04**	0.62 ±0.06	0.58 ± 0.05	0.62 ± 0.04	0.59 ± 0.05	0.60 ± 0.05	**0.69 ± 0.04**	0.50 ± 0.01	0.68 ± 0.05	-
HSE	**0.66±0.04**	0.60 ±0.03	0.56 ± 0.04	0.59±0.04	0.59±0.04	0.60±0.04	0.65±0.04	0.56±0.040	0.62 ± 0.05	≈ 1%
MMP	0.78±0.03	0.69 ±0.03	0.67± 0.02	0.68± 0.03	0.65 ± 0.02	0.68 ± 0.02	0.78 ± 0.02	0.51 ± 0.014	**0.80 ± 0.02**	
p53	**0.76 ± 0.03**	0.63 ±0.04	0.60 ± 0.04	0.68 ± 0.02	0.66 ± 0.03	0.65 ± 0.03	0.70 ± 0.03	0.54 ± 0.02	0.71 ± 0.03	≈ 5%

TABLE 3 REPORTED P-VALUES (UPTO FIVE DECIMAL PLACES) BETWEEN 3DGHK AND PK, GHK, RWK, SPK, WLK, GCN, GCN-ATTR.

Alt text: Table 3 shows the hypothesis testing results of 3DGHK with other models. P-values show that results for 3DGHK are statistically significant in comparison to other models except WLK and GCN-attr, as p-values for these cases are more than 0.05.

	3DGHK		3DGHK
PK	0.00002	GHK	0.00000
RWK	0.00009	SPK	0.00051
WLK	0.08587	GCN	0.00000
GCN-Attr	0.37902		

TABLE 4. COMPARISON BASED ON 3-HOP NEIGHBORS VERSUS 2-HOP NEIGHBORS IN OUR FRAMEWORK.

Alt text: Table 4 shows the decrease in accuracy of 3DGHK when restricted to 2-hop neighbours for dataset 1.

Dataset	with 3-hops	with 2-hops
1798	0.61±0.044	0.55 ±0.061
1843	0.75±0.065	0.67±0.062
2258	0.71± 0.045	0.59±0.046
2689	0.73±0.049	0.57±0.057
435008	0.58± 0.058	0.50±0.056
435034	0.64 ±0.036	0.60±0.035
463087	0.69 ±0.030	0.63±0.037
485290	0.67 ± 0.053	0.57±0.065
488997	0.62±0.049	0.55±0.071

TABLE 5. COMPARISON BASED ON 3-HOP NEIGHBORS VERSUS 2-HOP NEIGHBORS IN OUR FRAMEWORK.

Alt text: Table 5 shows the decrease in accuracy of 3DGHK when restricted to 2-hop neighbours for dataset 2.

Dataset	with 3-hops	with 2-hops
AhR	0.71 ± 0.024	0.65±0.028
AR	0.72 ±0.045	0.72± 0.037
AR.LBD	0.74±0.036	0.74±0.046
Aromatase	0.70 ± 0.026	0.67 ±0.026
ER	0.65 ± 0.02	0.63 ±0.026
ER.LBD	0.69 ± 0.032	0.68±0.033
PPAR.g	0.61 ± 0.042	0.61±0.053
ARE	0.65 ±0.021	0.63±0.028
ATAD5	0.63 ± 0.037	0.61 ±0.038
HSE	0.63±0.030	0.59±0.039
MMP	0.72±0.018	0.66±0.016
p53	0.67 ± 0.025	0.64 ± 0.030
