# Supplementary figures and images for: Efficient 3D kernels for molecular property prediction
Source: Bioinformatics. 2025 Jul 15;41(Suppl 1):i58–67. doi: 10.1093/bioinformatics/btaf208 (PMC12261455; doi:10.1093/bioinformatics/btaf208)

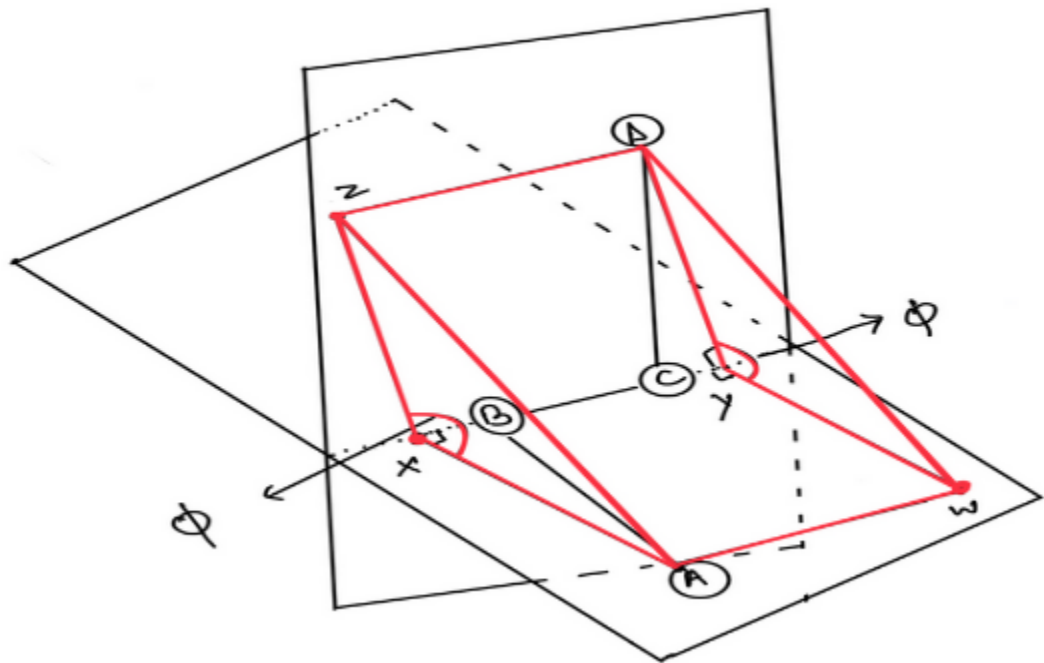

Supplement: btaf208_Supplementary_Data [file btaf208_supplementary_data.zip › btaf208_Supplementary_Data/Ankit.49.sup.1.fig.1.pdf]
